# RgIA4 Accelerates Recovery from Paclitaxel-Induced Neuropathic Pain in Rats

**DOI:** 10.3390/md18010012

**Published:** 2019-12-21

**Authors:** Peter N. Huynh, Denise Giuvelis, Sean Christensen, Kerry L. Tucker, J. Michael McIntosh

**Affiliations:** 1 School of Biological Sciences, University of Utah, Salt Lake City, UT 84112, USA; 2 Center for Excellence in the Neurosciences, University of New England, Biddeford, ME 04005, USA; 3 Dept. of Biomedical Sciences, College of Osteopathic Medicine, University of New England, Biddeford, ME 04005, USA; 4 George E. Whalen Veterans Affairs Medical Center, Salt Lake City, UT 84112, USA; 5 Department of Psychiatry, University of Utah, Salt Lake City, UT 84112, USA

**Keywords:** nicotinic, chemotherapy, paclitaxel, taxane, neuropathic pain, α9α10, conotoxin

## Abstract

Chemotherapeutic drugs are widely utilized in the treatment of human cancers. Painful chemotherapy-induced neuropathy is a common, debilitating, and dose-limiting side effect for which there is currently no effective treatment. Previous studies have demonstrated the potential utility of peptides from the marine snail from the genus *Conus* for the treatment of neuropathic pain. α-Conotoxin RgIA and a potent analog, RgIA4, have previously been shown to prevent the development of neuropathy resulting from the administration of oxaliplatin, a platinum-based antineoplastic drug. Here, we have examined its efficacy against paclitaxel, a chemotherapeutic drug that works by a mechanism of action distinct from that of oxaliplatin. Paclitaxel was administered at 2 mg/kg (intraperitoneally (IP)) every other day for a total of 8 mg/kg. Sprague Dawley rats that were co-administered RgIA4 at 80 µg/kg (subcutaneously (SC)) once daily, five times per week, for three weeks showed significant recovery from mechanical allodynia by day 31. Notably, the therapeutic effects reached significance 12 days after the last administration of RgIA4, which is suggestive of a rescue mechanism. These findings support the effects of RgIA4 in multiple chemotherapeutic models and the investigation of α9α10 nicotinic acetylcholine receptors (nAChRs) as a non-opioid target in the treatment of chronic pain.

## 1. Introduction

**Figure 1:**
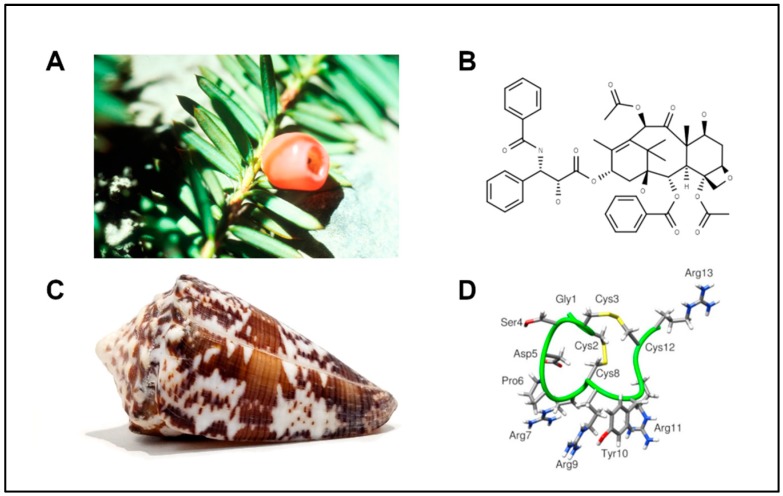
Natural sources of potential therapeutics. (**A**) The needles and berries of a Pacific yew, characteristic of the tree from which paclitaxel was originally extracted. (**B**) Chemical structure of paclitaxel. (**C**) Shell of the worm-hunting snail, *Conus regius*, from which RgIA was originally characterized. (**D**) Structure of the short 13-amino-acid peptide RgIA isolated from *Conus regius*. Image sources: (**A**) Yew Needles and Berries, National Cancer Institute Visuals Online, no. AV-9100-3761. (**C**) *Conus regius* photograph by Peter Huynh.

Neuropathic pain is a type of chronic pain that stems from the damage or disease of the sensory nervous system that affects an estimated 6.9–10% of the general population [1]. This type of pathological pain has many causes, including traumatic nerve injury, metabolic disorders such as diabetes, and chemical damage from chemotherapeutics [2]. Paclitaxel (Figure 1) is an anti-cancer drug of the taxane family, initially extracted from the bark of the Pacific yew (*Taxus brevifolia*), and perhaps the most well-known natural-product chemotherapeutic. It is used as a first-line treatment for breast, ovarian, and non-small cell lung cancers [3]. Several chemotherapies from different drug families, including taxane- and platinum-based drugs, have been characterized to produce painful neuropathies as a side effect. In the context of cancer therapies, these side effects can be more than disruptive as they are often dose-limiting in treatment regimens [4].

Patients have exhibited several types of neuropathies, including numbness, chronic pain, and allodynia (painful hypersensitivity) to mechanical or thermal stimuli [5]. Amongst patients, however, the type, duration, and severity of neuropathy can vary [6]. While the prevalence and manifestations of paclitaxel-induced neuropathy have been well documented, the underlying mechanisms are still being characterized. Several adjuvant treatments have been used in an attempt to combat the effects of chemotherapy-induced peripheral neuropathy (CIPN). However, there are currently no FDA-approved medications for the prevention or treatment of CIPN.

Cone snails have historically displayed a repertoire of therapeutic molecules. The venomous marine gastropods of the genus *Conus* are a diverse collection of snails that have developed complex hunting and envenomation strategies. The venoms of these snails contain hundreds of unique peptides, and the contents of these venoms can also change in response to defensive or predatory stimuli [7]. Cone snails have refined a suite of bioactive peptides that can exquisitely and potently discriminate among receptors involved in neurotransmission. These targets include G-protein-coupled receptors and voltage- and ligand-gated ion channels [8]. The chemical arsenal of each snail also contains bioactive compounds that have been characterized as prey-endogenous mimetics, such as the insulin-like peptide used by *Conus geographus*, which more closely resembles fish insulin than its own [9]. The discovery of this molecular mimicry strategy spurred the characterization of several other hormone/neuropeptide-like peptides in the venom repertoire of these snails [10]. 

Currently, there are an estimated 750+ species of cone snails whose venom components include small, disulfide-rich peptides that have been classified into at least 28 different superfamilies. These superfamilies are primarily subdivided by their conserved cysteine frameworks. Further characterization of cone snail venom ducts have also revealed the presence of small molecules that contribute to their venom activity [11]. Previous estimates of 50–200 unique compounds per venom have been expanded nearly 10-fold with the advancement of mass spectroscopy and venomics techniques. These estimates yield a collection of greater than 1 million potential lead peptide compounds to be characterized, less than 1% of which (~10,000) have been partially characterized [12,13]. While advancements in genomics, proteomics, and transcriptomics have rapidly accelerated the discovery of conotoxin peptides, the structural and pharmacological characterization has been rate-limiting. Notably, the ω-conotoxin MVIIA (Prialt® (ziconotide)), a non-opioid drug for intractable pain, remains the only FDA-approved conotoxin-based drug to date [14,15]. The majority of bioactive wealth from cone snail venoms awaits to be characterized. The classification and therapeutic applications of conotoxins have been reviewed in considerable detail [7,8,10,12,14,15,16,17,18,19,20].

Among the smallest of the peptides found in *Conus* venoms are the α-conotoxins, which competitively inhibit nicotinic acetylcholine receptors (nAChRs) [16]. α-conotoxins are typically 13–20 amino acids in length and disulfide-constrained. nAChRs are pentamers typically assembled from α and non-α subunits including α1–α10, β1–β4, and γ, δ, and ε in coordinated distributions. There are also homomeric assemblies consisting only of α-subunits. Together, these subunit combinations yield a large diversity of potential nAChR subtypes. 

Previously, we have reported the specific block of α9α10 nAChRs by the α-conotoxin RgIA [21,22]. This 13-amino-acid peptide, isolated from the worm-hunting snail *Conus regius*, blocked the rodent α9α10 nAChR with high potency; however, it was found to be approximately 300-fold less potent on the human receptor due to a single amino-acid substitution in the α9 nAChR subunit [23]. The second-generation synthetic analog, RgIA4, was engineered to close this affinity gap across the rodent and human α9α10 nAChRs (IC_50_ rat = 0.9 nM; IC_50_ human = 1.5 nM), and has also been shown to effectively prevent oxaliplatin-induced pain in rats and mice [24,25]. These findings are consistent with previous behavioral and cellular studies that demonstrate native RgIA can prevent the development and/or progression of neuropathic pain in chronic constriction injury and oxaliplatin-induced injury [26,27,28]. RgIA and several other α-conotoxins from worm-hunting *Conus* species act on ancestral nAChRs such as α9, α10, or α7-containing subtypes. While these α-conotoxins may incapacitate their native prey, their effect on higher-order species is more nuanced, since the target receptors may play roles outside the neuromuscular junction [29]. Here, we show that, in addition to preventing oxaliplatin-derived neuropathic pain, RgIA4 is efficacious in accelerating recovery from paclitaxel-induced neuropathic pain in rats.

## 2. Results

### 2.1. RgIA4 Accelerates Recovery from Paclitaxel-Induced Allodynia in Sprague Dawley Rats

**Figure 2:**
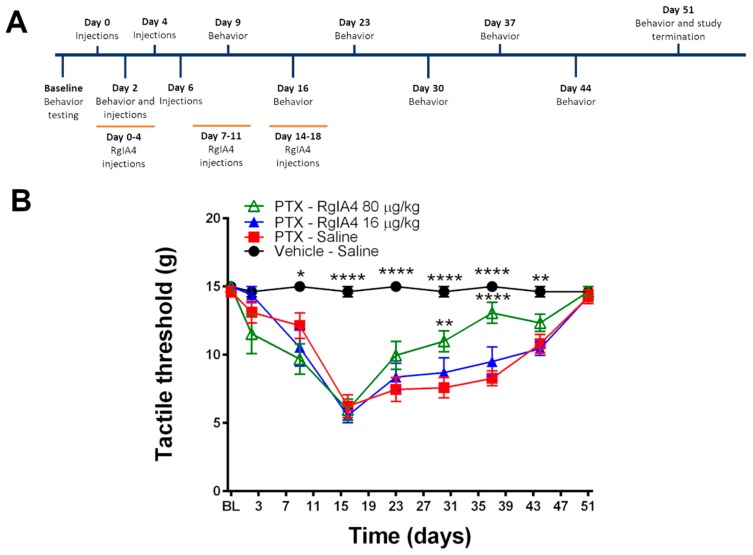
RgIA4 accelerates recovery from mechanical allodynia. (**A**) Study timeline. Sprague Dawley (SD) rats (*n* = 8) were treated with either vehicle (intraperitoneally (IP))-saline (subcutaneously (SC)), 8 mg/kg paclitaxel (PTX) (IP)-saline (SC), 8 mg/kg paclitaxel (IP)-RgIA4 (80 μg/kg; SC), or 8 mg/kg paclitaxel (IP)-RgIA4 (16 µg/kg; SC). Animals were tested for behavior prior to the first dose of paclitaxel (baseline (BL)) and over the course of 51 days. (**B**) Testing results from Von Frey assay. Results are expressed as tactile threshold values in grams (g). Black circles: vehicle-saline; red squares: PTX-saline; blue triangles: PTX-RgIA4 (16 µg/kg); hollow green triangles: PTX-RgIA4 (80 µg/kg). Mean +/- SEM are indicated. Two-way ANOVA was conducted followed by Bonferroni’s multiple comparison test, alpha = 0.05. Asterisks denote a significant difference from the PTX-Saline curve (* *p* < 0.05, ** *p* < 0.01, and **** *p* < 0.0001).

Semisynthetic taxanes, including paclitaxel, are first-line treatments for the most common solid tumors, but taxane-induced peripheral neurotoxicity is a frequent dose-limiting side effect. Sprague Dawley (SD) rats are commonly used to model paclitaxel-mediated CIPN due to the consistent induction of mechanical and thermal allodynia and ease of behavioral readouts compared to mice [30,31,32]. A clinical formulation of paclitaxel was chosen in order to induce a longer-lasting neuropathic pain effect [31]. Adult SD rats were injected four times intraperitoneally (IP) with paclitaxel (2.0 mg/kg) on days 0, 2, 4, and 6, for a total dosage of 8.0 mg/kg. Over the course of the paclitaxel injections and over the next 12 days, rats were also administered daily subcutaneous (SC) injections of RgIA4 at 16 and 80 µg/kg five days per week (days 0–18) (Figure 2A). To assay for mechanoreceptive properties of paclitaxel-induced neuropathic pain, SD rats were tested through hindpaw von Frey analysis. 

Compared to vehicle-injected animals, paclitaxel-injected rats showed a robust, painful sensitization to previously non-painful mechanical stimuli by day 9 which persisted through day 44 (Figure 2B). These effects peaked on day 16, where the paclitaxel (PTX)-saline-treated rats exhibited a pain response from the Von Frey filaments, withdrawing or licking their paw at a mean threshold of 6.2 g, compared to the vehicle-saline-treated rats, whose threshold was at a mean force of 14.6 g. Co-administration of RgIA4 (16 or 80 μg/kg; SC) did not produce analgesic effects during the induction of neuropathic pain (days 0–16). 

By day 23, the paclitaxel-treated groups began a modest recovery from their mechanical allodynia. However, by day 31, rats treated with 80 μg/kg of RgIA4 showed accelerated recovery from the paclitaxel-induced hypersensitivity, reaching significance compared to the paclitaxel-saline-treated rats (*p* < 0.01, as determined by two-way ANOVA followed by Bonferonni’s multiple comparison test). The rate of recovery by the RgIA4 (80 μg/kg)-treated rats maximally outpaced all other groups by day 37 (*p* < 0.0001). Remarkably, this therapeutic effect was first reached 12 days after treatment with RgIA4 had been stopped. 

**Figure 3:**
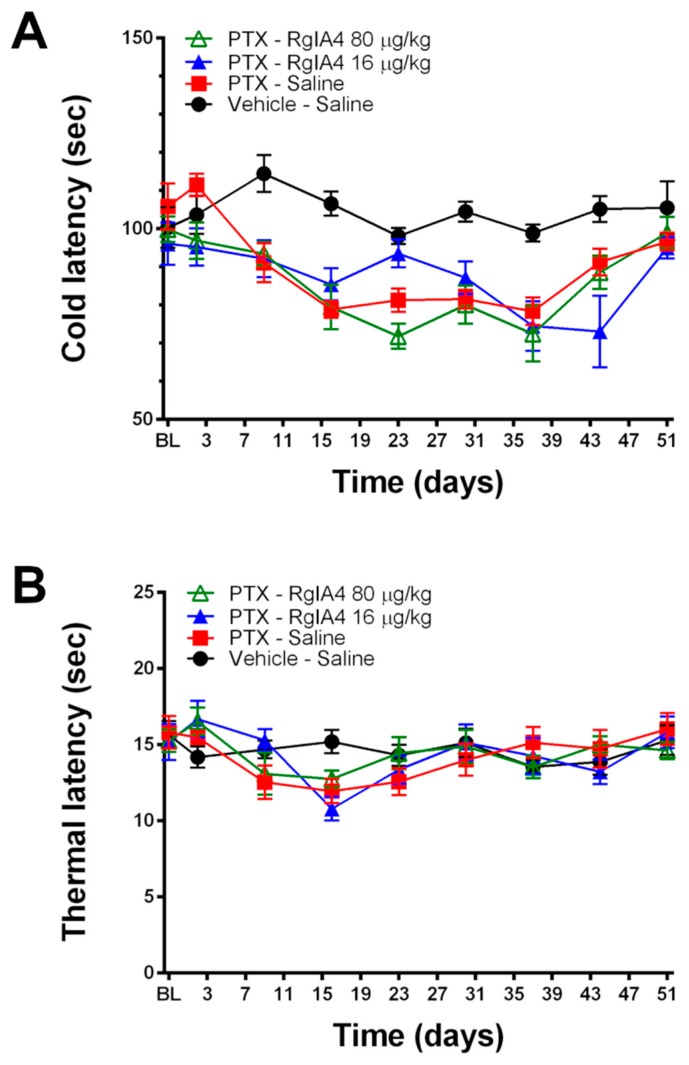
RgIA4 did not reverse cold allodynia and paclitaxel did not induce heat allodynia under these conditions. Testing results from (**A**) cold plate and (**B**) Hargreaves assays following treatment of SD rats (*n* = 8 per group, unless otherwise noted) with a total dose of 8 mg/kg paclitaxel with or without RgIA4 at 80 or 16 μg/kg. Animals were tested for behavior from the first dose of paclitaxel (BL) over the course of 51 days. Results are expressed in (**A**) cold and (**B**) thermal latency times in seconds (s). Black circles: vehicle-saline; red squares: PTX-saline; blue triangles: PTX-RgIA4 (16 μg/kg); hollow green triangles: PTX- RgIA4 (80 μg/kg). Mean +/- SEM are indicated.

**Figure 4:**
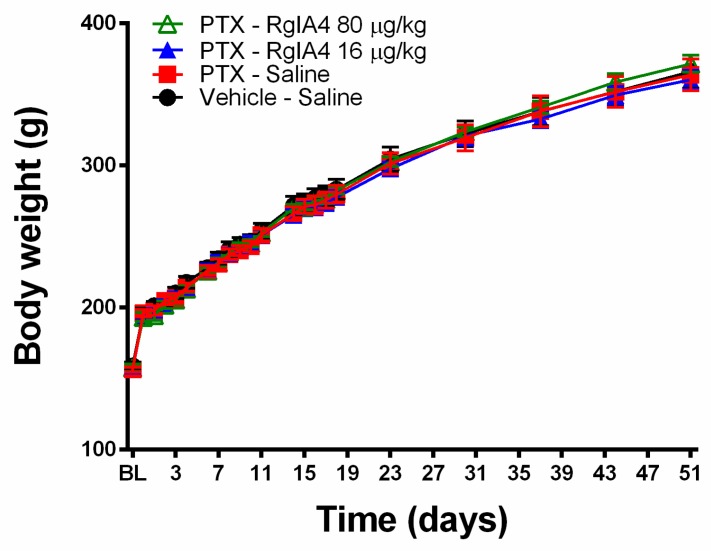
Neither paclitaxel nor RgIA4 significantly affected body weight over time. Changes in rat body weight (in g) are indicated following treatment with a total dose of 8 mg/kg paclitaxel, with or without RgIA4 at 80 or 16 μg/kg. Readings were taken from cohorts of *n* = 8 rats per group, unless otherwise specified. Black circles: vehicle-saline; red squares: PTX-saline; blue triangles: PTX-RgIA4 (16 μg/kg); hollow green triangles: PTX-RgIA4 (80 μg/kg). Mean +/- SEM are indicated.

Further analysis of thermoreceptive properties revealed no ameliorative effects of RgIA4 at either dosage as determined by a cold plate assay (Figure 3). Neither paclitaxel nor RgIA4 affected heat allodynia, as measured by the Hargreaves test, nor the weight gain of the animals over the course of the experiment (Figure 4). The dosages of 80 and 16 μg/kg were chosen based on previously characterized regimens that effectively produced relief in neuropathic pain models in mice and rats [24,25]. These dosages have been previously reported to produce no adverse effects in motor coordination nor CNS function based on rotarod and Irwin tests [25].

### 2.2. Paclitaxel Did Not Induce Mechanical Allodynia in C57BL/6J Mice

Rats have been widely used in models of neuropathic pain. When mice are used, the C57BL/6J mouse is perhaps the most commonly used inbred strain; its entire genome has been sequenced, and a wide variety of transgenics are available. The effectiveness of inducing CIPN pain in C57BL/6J mice with paclitaxel, however, has been reported with varying levels of success. Some previous reports showed robust induction of CIPN by paclitaxel in C57BL/6J mice, while others were not able to create this pain state [32,33]. The diverse genetic background of inbred mouse strains has historically resulted in variable levels of CIPN severity. This has been documented across several strains of mice with both paclitaxel and oxaliplatin as the agent of induction [32,34]. Additional, early genome-wide association studies (GWASs) have suggested genetic predispositions to CIPN pain [35]. This is also reflective of clinical reports, where the severity and duration of painful neuropathy varies between patients [4].

In our experiments, administration of C57BL/6J mice with 2.0 mg/kg of paclitaxel (IP) on days 0, 2, 4, and 6 did not produce a statistically significant change in mechanical allodynia as measured by Von Frey (Supplemental Figure S1). As a measure of neurophysiological integrity, the velocity and amplitudes of sensory nerve action potentials (SNAPs) are commonly used as a readout in both clinical and preclinical settings [36,37]. Previous studies have reported the reduction of action potential amplitude in patients receiving paclitaxel as the duration of their treatment regimen progressed [38]. In our cohort of C57/BL6 mice, there was no change in observed nerve conduction velocities (NCVs) nor amplitudes of SNAPs in the tail between treated and untreated mice (Supplemental Figure S2). Due to the lack of robust induction under these conditions, we did not continue with further behavioral tests in C57BL/6J mice.

## 3. Discussion

### 3.1. α9α10 nAChRs as a Target for Pain Treatment

RgIA4 has been previously shown to prevent the induction of CIPN pain by the platinum-based chemotherapeutic, oxaliplatin [24,25]. Since chemotherapeutic agents work by different mechanisms of action to inhibit tumor growth, we wished to assess the activity of RgIA4 in taxane-induced neuropathic pain [39]. Taxanes are effective treatments for breast cancer; however, neuropathic pain is a common side effect. After two years of treatment, over 40% of women indicated that they still experience neuropathy symptoms with compromised long-term quality of life [40,41]. There are currently no recommended agents for the prevention of taxane-induced neuropathic pain. The positive outcome for RgIA4 indicates broader applicability of α9α10 nAChR antagonists for preventing symptoms of CIPN.

In this study, the repeated and intermittent administration of clinically-formulated dosages of paclitaxel produced a robust mechanical allodynia which was consistent with previous reports in both humans and rodents [5]. A previously-observed coasting phenomenon was also present in which symptoms could continue and even intensify after cessation of treatment. In this study, symptoms peaked in intensity on day 16, ten days after the last administration of paclitaxel; a similar phenomenon has been reported in clinical settings and successfully reproduced in rodent models [31,42]. The administration of RgIA4 five days per week for three weeks successfully accelerated the recovery from paclitaxel-induced mechanical allodynia in a dose-dependent manner. The effects of RgIA4 only became evident after repeated dosages and did not reach significance until day 30, approximately 12 days after the RgIA4 administration had been discontinued. This delay in efficacy is consistent with our previous reports of both native RgIA in chronic constriction injury models of neuropathic pain and RgIA4 in oxaliplatin-mediated neuropathic pain. The time course of symptom relief is consistent with a disease-modifying effect of RgIA4 rather than just a pain-masking effect [24,25,26]. In future studies, it will be of interest to assess the effects of RgIA4 that are administered over a longer time frame.

The diversity of cone snails and their venom components have provided a rich pharmaceutical cornucopia of neuroactive compounds. Previously, α-conotoxins such as Vc1.1 and RgIA have been characterized to produce anti-pain effects in rodent models of chronic pain, as have been members of the ω-conotoxin family such as MVIIA [28,43]. MVIIA is commercially available as Prialt® (ziconotide) for the treatment of intractable pain and acts by blocking N-type calcium channels in the CNS. Notably, N-type calcium channels may be inhibited by stimulation of γ-aminobutyric acid type-B (GABA_B_) receptors or µ-opioid receptors [44,45,46]. The stimulation of GABA_B_ receptors has been proposed as the mechanism of action for α-conotoxins Vc1.1 and RgIA [47,48,49,50,51,52]. We note, however, that the RgIA analog used in the present study, RgIA4, does not have GABA_B_ or µ–opioid activity [24,25]. 

It is noteworthy that the analgesic effects of RgIA4 do not become apparent until the discontinuation of treatment. Previous studies of RgIA and RgIA4 have indicated that full analgesic effects may occur after several weeks of treatment [24,25,26]. This efficacy time course is consistent with a disease-modifying effect that we observed in oxaliplatin-induced neuropathic pain, where RgIA4-treated animals demonstrate anti-allodynic benefits for several weeks after the discontinuation of RgIA4 treatment [24]. This suggests that RgIA4 affects the progression of chronic pain development and recovery, yielding pain relief even after the clearance of RgIA4 from circulation. While neuropathic pain has many different causes, a point of convergence in the progression of disease is neuroinflammation and peripheral nerve damage [2]. α9α10 nAChRs have not been shown to be involved in the neurotransmission of pain. However, these receptors have been shown to be expressed in peripheral immune cells and to affect the release of inflammatory cytokines in vitro and the infiltration of immune cells into perineural spaces following chronic constriction injury (CCI) [28,53,54]. While the exact mechanisms by which α9α10 nAChR antagonists exert their therapeutic effects have yet to be elucidated, it is likely that the long-term neuroprotective effects are, at least in part, influenced by neuroimmune-mediated mechanisms.

Other α9α10 antagonists, structurally unrelated to RgIA4, have also been shown to have analgesic activity in CIPN pain and other neuropathic pain models. Small molecule azaaromatic quaternary ammonium analogs selectively block α9α10 nAChRs [55]. The bis-analog, ZZ1-61c, prevented the development of vincristine-induced neuropathic pain [56]. The tetrakis-quaternary ammonium ZZ-204G was analgesic in formalin and CCI models of neuropathic pain [57]. In addition, an entirely different conotoxin peptide, αO-conotoxin GeXIVA, which blocks α9α10 nAChRs noncompetitively and lacks GABA_B_ agonist activity, also reverses oxaliplatin-induced neuropathic pain and CCI pain [58,59,60]. 

Conotoxins may be classified by gene superfamily and defined by their signal sequence in the prepropeptide region and their disulfide framework. The α-conotoxins are members of the A-superfamily, which are characterized as two-disulfide-bridged peptides, typically 13–19 amino acids in length, that selectively and competitively inhibit nAChRs [61]. By contrast, αO-conotoxin GeXIVA is from the O1-superfamily, which are three-disulfide-bridged peptides that typically inhibit voltage-gated ion channels [19]. This diverse group of antagonists supports the idea that α9α10 nAChRs are an effective target for reversing and accelerating recovery from neuropathic pain.

### 3.2. Chemotherapy-Induced Neuropathic Pain and Block of α9α10 nAChRs

Chemotherapeutics of the taxane, vinca-alkaloid, and platinum-based families have different mechanisms of action. Paclitaxel is a member of the taxane family and produces a robust anti-cancer effect by stabilizing microtubule polymers, effectively preventing the disassembly and progression of mitosis [62,63,64]. Vinca alkaloids also target microtubules, but in a fashion “opposite” to taxanes, do so by destabilizing microtubule formation [65]. By contrast, platinum-based drugs such as oxaliplatin primarily target DNA, creating DNA lesions and adducts, ultimately preventing cell replication [66]. Just as the mechanisms of action between taxanes, vinca alkaloids, and platinum-based drugs are different, the resulting neuropathologies also develop differently. Taxanes cause dysfunction to mitochondria and endoplasmic reticulum calcium signaling as a side effect of their microtubule disruption, whereas platinum-based drugs appear to alter surface ion-channel remodeling initially, and eventually lead to neuronal apoptosis after prolonged exposure [67,68,69]. Vincristine has also been shown to upregulate the surface expression of 5-hydroxytryptamine2A (5-HT_2A_)receptors in neurons in the dorsal horn and dorsal root ganglion (DRG), sensitizing nociceptors [70]. The development and progression of neuropathies between these three distinct classes of chemotherapeutics are multi-faceted, overlapping in certain aspects, and yet diverse in nature [71]. 

Painful neuropathies caused by oxaliplatin and paclitaxel share some commonalities of neuronal inflammation, altered ion-channel-expression and excitability of peripheral neurons, and dose-dependent severity [69]. However, while several cellular and molecular markers have been characterized in the development of these distinct neuropathies, the pathophysiology still remains poorly understood [72,73,74]. Previously, RgIA4 showed antinociceptive efficacy in oxaliplatin-treated rats with dosages as low as 0.128 μg/kg, compared to this study where effects did not become evident until 80 μg/kg [24,25]. In addition, RgIA4 showed a robust reversal of cold allodynia in these oxaliplatin models of CIPN. The reason for these differences is unknown. As such, the differential efficacy of RgIA4 in paclitaxel-treated animals, as compared to previously-reported oxaliplatin-treated animals, may provide a pharmacological tool that can help investigate the distinct, yet intertwining, disease progression between these two pathologies.

Despite the different mechanisms of neuropathology caused by distinct anti-cancer drug classes, the blockade of α9α10 nAChRs appears to be a potential convergent target for the prevention and/or treatment of neuropathic pain across several models. Separate from the α9α10 nAChR subtype, the closely-related homomeric α7 nAChR and the α74β2 nAChR subtypes have been implicated in the modulation of pain. Selective activation of α7 nAChRs with (R)-(-)-3-methoxy-1-oxa-2,7-diaza-7,10-ethanospiro[4.5]dec-2-ene sesquifumarate ((R)-ICH3) or PNU-282987, positive allosteric modulation with GAT107 or PNU-120596, and silent agonism with (R)-N-(4-Methoxyphenyl)-2-((pyridin-3-yloxy)methyl)pi-perazine-1-carboxamide Dihydrochloride (R-47) have shown efficacy in models of inflammatory and neuropathic pain [75,76,77,78]. Separately, the α4β2 nAChR agonist (R)-5-(2-azetidinylmethoxy)-2-chloropyridine (ABT-594), an analog inspired by the alkaloid epibatidine isolated from poison arrow frog, *Epipedobates tricolor*, has been shown to result in robust anti-pain effects [79,80]. ABT-594 moved forward into clinical trials but was halted due to adverse side effects.

While chemotherapies are diverse in their mechanisms of action, several drugs across different families have produced similar neuropathies. The use of natural products as a source of therapeutics has proven to be effective but with caveats. Harnessing the evolutionary refinement of these compounds has provided a rich collection of highly specific pharmacological tools. The discovery of paclitaxel was a fortuitous hit from a concerted screening of natural products coordinated by the United States National Cancer Institute looking specifically for compounds that would be efficacious against cancers [3]. Although one of the most widely used chemotherapeutics, the disruptive side effects of paclitaxel are evident. The use of a cone-snail-derived compound as an effective adjuvant treatment highlights the versatility of natural compounds and the importance of continuing the concerted screening and characterization of these molecules for therapeutic applications. The observed efficacy of α9α10 nAChR antagonists in preventing CIPN pain from taxane, platinum-containing, and vinca-alkaloid families of chemotherapeutics suggests a common juncture in the mechanisms of CIPN development across several structural classes of compounds. RgIA4 may be a promising tool in both the treatment of CIPN pain through this mechanism and further characterization of this non-opioid pathway of pain treatment as a molecular probe.

## 4. Materials and Methods 

### 4.1. Animals

Male Sprague Dawley rats (Envigo) weighing 200–300 g were used in all rat experiments. Rats were housed in groups of two rats per cage with food and water available ad libitum. Animals were maintained in a temperature- and humidity-controlled animal colony maintained on a 12-h light/dark cycle (lights on at 07:00). Male C57BL/6J mice (Jackson Laboratories) were used in all mouse experiments. Mice were housed in groups of four or five per cage with food and water available ad libitum and on the same light cycle. All rat studies were conducted under the approval of the Institutional Animal Care and Use Committee (IACUC) of the University of New England, protocol number 042617-001. All mice experiments were conducted under the approval of the University of Utah IACUC, protocol number 17-08002. The Animal Care and Use Programs and Facilities of the University of Utah are accredited by the Association for Assessment and Accreditation of Laboratory Animal Care (AAALAC) International.

### 4.2. Drug Solutions and Injections

A clinical formulation of paclitaxel was employed. Paclitaxel USP, extracted from Taxus X media ‘Hicksii’ (Hospira, IL, USA), came in solution at a concentration of 6 mg/mL. The vehicle used for dilution consisted of 1 part 1:1 Cremophore:EtOH mixture and 2 parts 0.9% saline, and 2 mg/mL sodium citrate. Rats were given a dose of 2 mg/kg (IP) every two days for a total of 4 doses, at a volume of 1 mL/kg body weight. RgIA4 was dissolved in 0.9% saline and injected (SC) once per day, 5 days a week for a total of 15 doses at 1 mL/kg body weight for rats and 4 mL/kg body weight for mice [25]. Upon completion of animal injections, material underwent HPLC analysis to verify its composition and integrity.

### 4.3. Von Frey Assay

Tactile allodynia was quantified by measuring the hindpaw withdrawal threshold to Von Frey filament stimulation, using the up-down method previously reported [81,82]. Throughout the study, experimenters were blinded to the identity of the injected compound. Rats were divided into treatment groups of *n* = 8 unless otherwise specified. Animals were placed in a clear Plexiglass chamber and allowed to habituate for 15–60 min. Touch-Test filaments (North Coast Medical, Morgan Hill, CA, USA) were used for all testing. For rats, the 2.0 g (4.31) filament was used to start. Clear paw withdrawal, shaking or licking was considered a positive or painful response. This up-down method was stopped four measures after the first positive response. The withdrawal threshold was calculated using the up-down Excel program generously provided by Dr. Michael Ossipov (University of Arizona, Tucson, AZ, USA). The filament range for rats was 3.61, 3.84, 4.08, 4.31, 4.56, 4.74, 4.93, 5.18.

### 4.4. Plantar Test

Thermal allodynia was quantified by measuring the hindpaw withdrawal latency to noxious radiant heat application in unrestrained animals using the plantar test apparatus (rats: Ugo Basile), as previously reported [83]. Throughout the study, experimenters were blinded to the identity of the injected compound. Rats were divided into treatment groups of *n* = 8 unless otherwise specified. Briefly, animals were placed in a clear Plexiglass chamber and allowed to habituate for 15–60 min. A radiant heat source was then focused on the plantar surface of the hindpaw with the paw withdrawal time automatically determined. The intensity of the heat source was adjusted so that baseline latency was approximately 15 s. A test cutoff time of 30 s was observed to avoid tissue damage. 

### 4.5. Cold Plate

Rats underwent cold plate testing to determine if any sensitivity to cold developed following paclitaxel injection. Throughout the study, experimenters were blinded to the identity of the injected compound. Rats were divided into treatment groups of *n* = 8 unless otherwise specified. A hot/cold plate (Ugo Basile) was used and cooled to 5 °C. To help achieve reliable results a layer of distilled water coated the cold plate. Each rat was placed on the plate, one at a time. The time it took the rat to lick or flick a hind paw or jump to escape the plate was recorded to the nearest 0.1 s. A 5 min cutoff time was used to remove the rat from the plate if they had not yet shown a response to avoid tissue damage.

### 4.6. Statistical Analysis

In the rat assays, dose- and time-response curves were constructed for each pain model. Mean and standard errors of the mean were calculated. To determine statistical significance either an unpaired *t*-test or two-way ANOVA was performed followed by a Bonferroni posttest using GraphPad Prism software version 6.07 for Windows, GraphPad Software, La Jolla, CA, USA. Significance was established at the *p* < 0.05 level. 

## Supplementary Materials

The following are available online at https://www.mdpi.com/1660-3397/18/1/12/s1, Figure S1: Paclitaxel did not induce mechanical allodynia in C57BL/6J mice, Figure S2: Tail synaptic nerve action potential (SNAP) conduction velocity was not affected by paclitaxel.

## Abbreviations

The following abbreviations are used in this manuscript:

ANOVA	Analysis of variance
CCI	Chronic constriction injury
CIPN	Chemotherapy-induced peripheral neuropathy
CNS	Central nervous system
GWAS	Genome-wide association study
IP	Intraperitoneal
nAChR	Nicotinic acetylcholine receptor
NCI	National Cancer Institute
NCV	Nerve conduction velocity
PTX	Paclitaxel
SC	Subcutaneous
SD	Sprague Dawley
SEM	Standard error of the mean
SNAP	Sensory nerve action potential
