# Supplementary material for: RgIA4 Accelerates Recovery from Paclitaxel-Induced Neuropathic Pain in Rats
Source: Mar Drugs. 2019 Dec 21;18(1):12. doi: 10.3390/md18010012 (PMC7024385; doi:10.3390/md18010012)
Supplement: Supplementary file 1 [file marinedrugs-18-00012-s001.pdf]

marine drugs

Supplemental figures, materials and methods

S1. Mouse von Frey assay

Paclitaxel was diluted in saline (0.9% sodium chloride) at 0.5 mg/mL. C57BL/6J mice were injected intraperitoneally (IP) with either paclitaxel (2 mg/kg) or saline. Mice were treated with paclitaxel every two days for a total of four administrations, yielding a cumulative dose of 8 mg/kg paclitaxel. Behavioral assays were performed prior to the first administration of paclitaxel to establish baseline, 24 hours after the last injection, and every 7 days thereafter. In mice, the von Frey assay was performed using a "MouseMet" electronic von Frey apparatus (Topcat Metrology Ltd., Little Downham, Ely Cambridgeshire, CB6 2TY, UK). The MouseMet device uses a rotary-force transducer to reduce tremors and force-irregularities from the operator. Briefly, mice were placed in elevated holding chambers with a barred floor and allowed to acclimate until investigative tendencies have ceased, approximately 5 minutes. Each mouse was tested 3x times per session. The probe is applied to the hindpaw and force is steadily increased until the animal releases from the barred floor and withdraws its paw. The MouseMet reads out a rate of force application with the curve maximum representing the force applied in grams (g). Mean and SEMs are reported and statistical significance was determined by the unpaired two-tailed t-test followed by the Sidak multiple comparison method. Researchers were blinded to the identity of the injected compounds for the duration of the study.

S2. Synaptic nerve action potential (SNAP) conduction velocity

Briefly, C57Bl/6J mice were assessed for sensory nerve action potential (SNAP) conduction velocity on days 21 through 23 as a terminal study, 24 hours post-injection. Mice were anesthetized using the EZ-7000 Classic System isoflurane vaporizer (E-Z Systems Inc., Palmer, PA, USA) at 5% and maintained at 1.5% for the duration of the analysis. The tail was immobilized onto a custom temperature-regulated Peltier system and two pairs of recording electrodes were inserted into the tail 20 mm apart. A pair of stimulating electrodes was placed 20 mm distally to the recording electrodes, and the ground electrode was inserted medially between the stimulating electrodes and first set of recording electrodes. Stimuli were applied at 10 ms into the recording window using a Model 2200 analog stimulus isolator (A-M Systems Sequim, WA, USA) and acquisition of SNAPs were collected using two battery-operated Grass Model P55 pre-amplifiers (Grass Instruments, West Warwick, RI, USA). Both devices were managed through a custom virtual instrument designed and run in the graphical programming language LabView (National Instruments, Austin, TX) [64]. Mean and SEMs are reported and statistical significance was determined by the unpaired two-tailed t-test. Researchers were blinded to the identity of the injected compounds for the duration of the study.

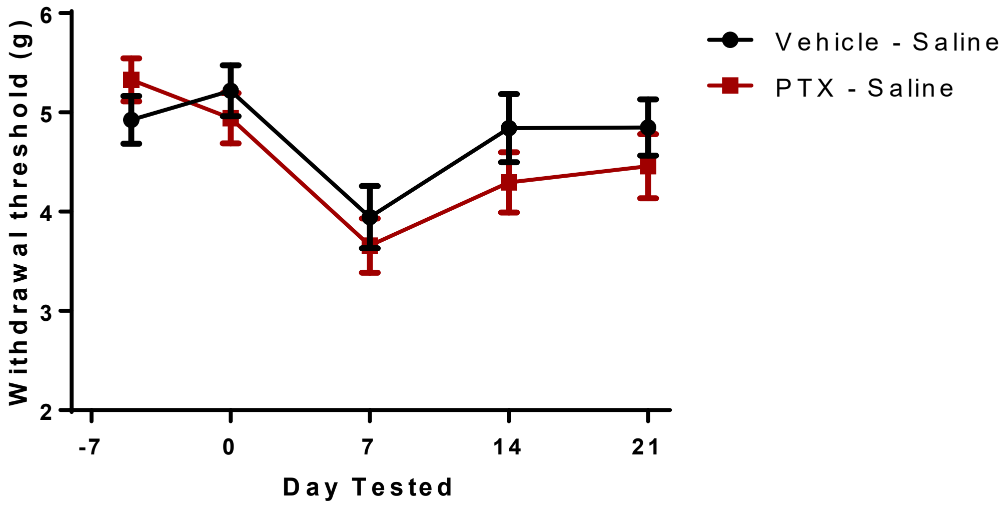

Figure S1. Paclitaxel did not induce mechanical allodynia in C57BL/6J mice. Mechanical allodynia was measured in naïve mice using an electronic von Frey apparatus as described in *Materials and Methods*, and longitudinally measured in the same mice after treatment. The mice (n=8) received four sequential treatments of paclitaxel on days 0, 2, 4, and 6, yielding a total dose of 8 mg/kg paclitaxel (IP) and saline (SC), or with vehicle (IP) and saline (SC). Mice were assayed prior to their first administration of paclitaxel, followed by a repeated test every 7 days for 21 days. Results are expressed in tactile threshold values in grams (g). Black circles: vehicle/saline and red squares: paclitaxel/saline. Mean +/- SEM are indicated. An unpaired two-tailed t-test followed by the Sidak multiple comparison test was run to determine significance.

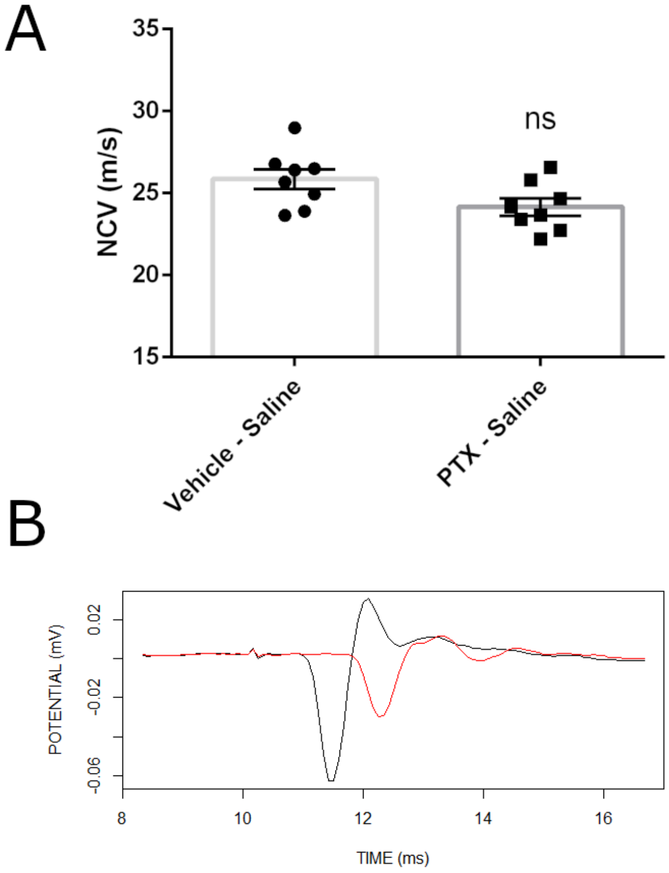

Figure S2. Tail synaptic nerve action potential (SNAP) conduction velocity was not affected by paclitaxel in C57BL/6J mice. A. SNAP nerve conduction velocities (NCVs) were collected as a terminal study on days 21-23. Each point represents the readings from a single mouse (n=8 per group). Black circles: vehicle/saline and red squares: paclitaxel/saline. Readings are expressed in meters/second. Mean velocity ± SEM are indicated. Analysis by an unpaired two-tailed t-test is reported. **B.** A representative trace of two SNAPs measured 20 mm apart. The electrical stimulus was applied at 10 ms. Readings are expressed in millivolts (mV) plotted in relation to time (ms).
